# Characterization of women with cervical cancer assisted at Inca by histological type

**DOI:** 10.11606/s1518-8787.2019053001218

**Published:** 2019-09-27

**Authors:** Suelem do Rozario, Iléia Ferreira da Silva, Rosalina Jorge Koifman, Ilce Ferreira da Silva

**Affiliations:** I Fundação Oswaldo Cruz. Escola Nacional de Saúde Pública Sérgio Auroca. Programa de Saúde Pública e Meio Ambiente. Rio de Janeiro, RJ, Brasil; II Fundação Oswaldo Cruz. Escola Nacional de Saúde Pública Sérgio Auroca. Departamento de Epidemiologia e Métodos Quantitativos em Saúde. Rio de Janeiro, RJ, Brasil

**Keywords:** Uterine Cervical Neoplasms, epidemiology, Papanicolaou Test, classification, Reproductive History, Risk Factors, Socioeconomic Factors

## Abstract

**OBJECTIVE:**

To determine the distribution of sociodemographic, reproductive, clinical and lifestyle habits in the cohort of women diagnosed with cervical cancer, assisted at Inca between 2012 and 2014, according to the histological type.

**METHODS:**

Retrospective observational study of a hospital cohort of 1,004 women diagnosed with cervical cancer. Data were obtained from the Inca hospital cancer registry, physical and electronic records.

**RESULTS:**

The most frequent histological type was squamous cell carcinoma (83.9%). Approximately 70% of the women aged more than 40 years. The study includes non-white women (67.4%), with less than 8 years of education (51.9%), with onset of sexual activity up to 16 years of age (40.7%), who were pregnant before (95.5%), with more than one pregnancy (82.9%), and more than two children (52.7%); 45.8% of the women were smokers or former smokers. Cervical adenocarcinoma was positively associated with earlier staging (IA-IIA) (OR = 1.79; 95%CI 1.03–3.13), as well as women with ≥ 12 years of education (OR = 6.30; 95%CI 1.97–20,13), who had no children (OR = 3.81; 95%CI 1.20 – 12,08) or who had up to two children (OR = 1.74; 95%CI 1.05 – 2,87).

**CONCLUSIONS:**

The difference between histological types is highlighted, suggesting that women with cervical adenocarcinoma may represent a distinct clinical entity of cervical neoplasia, which may require different approaches from those used in squamous cell carcinoma.

## INTRODUCTION

Cervical cancer is one of the main causes of cancer death among women worldwide, especially in developing countries, where 83% of new cases and 86% of deaths occur. According to estimates of the International Agency for Research on Cancer (IARC), in 2012 the incidence and mortality rates of the disease in Brazil were 14/100,000 and 6.8/100,000 inhabitants, respectively^1^.

The implementation of screening and treatment programs of cervical cancer precursor lesions has declined both the incidence and mortality rates of cervical squamous cell cancer in the last 50 years in developed countries^2,3^and, in most recent decades, in some developing countries^4,5^. This neoplasia may present different histological types, of which squamous cell carcinoma (SCC) is the most frequent (80%), while cervical adenocarcinoma (AC) and adenosquamous carcinoma (ASC) represent 10-15% of the cases^6^. However, an increase in the incidence of AC was observed in developed countries, especially in women aged between 20 and 40 years^2^.

Human papillomavirus (HPV) infection is the necessary but not sufficient cause of uterine cervix cancer, and exposure to cofactors is required for the tumor phenotype to occur^4,7^. However, some risk cofactors are specifically associated with AC. Evidence suggested that, besides immunosuppression^8^ and factors related to sexual behavior^9^, which are common to both histological types, use of oral contraceptive pills (OCP)^4,9^, obesity^10^and nulliparity^9^ are related to this subtype.

The low frequency of this neoplasia, especially cervical adenocarcinoma, in developed countries limited the evaluation of the associated factors. Thus, developing countries, which present a high incidence of this cancer, would have a more robust analysis opportunity due to the possibility of a larger sample size. Nevertheless, a few Brazilian studies on the profile of the distribution of factors associated with cervical cancer according to the histological type, the two largest studies were performed based only on the information provided by the Hospital-Based Cancer Registries (HBCR) and none of the studies estimated which factors were associated with the histological type^11,12^. Thus, the aim of this study was to determine the distribution of sociodemographic, reproductive, clinical and lifestyle characteristics in the cohort of women diagnosed with cervical cancer, assisted at Inca between 2012 and 2014, according to the histological type.

## METHODS

This study is a subproject of the research entitled “Evaluation of the waiting time in the therapeutic management and its effects on the survival of women diagnosed with cervical cancer in a hospital cohort at Inca II,”, approved by the Research Ethics Committee of Inca in October 2015.

A descriptive and exploratory observational study was conducted in a cohort of women with primary diagnosis of cervical cancer enrolled and treated in a center that concentrates all cases of gynecological cancer of Inca, between July 2012 and October 2014.

The study population consisted of the universe of primary cervical cancer cases – coded as C53.0 by the 10th revision of the International Classification of Diseases (ICD-10) – with histopathological confirmation of the following histological types: squamous, adenosquamous, and adenocarcinoma. The exclusion criteria were cases of carcinoma of the uterine cervix *in situ* and cases that started treatment outside of Inca.

**Figure 1:**
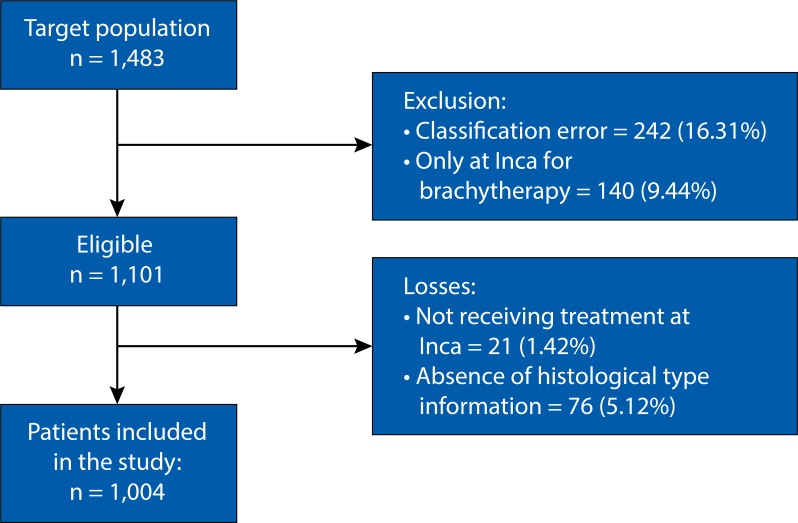
Flow of selection of women for the study.

Based on the identification of cases classified in HBCR, the review of physical and electronic records and histopathological reports was performed. From July 2012 to October 2014, 1,483 women with cervical cancer enrolled the study. The number of 242 women (16.31%) were excluded because they presented histological type error, 140 (9.44%) because they came from another health institution just for the brachytherapy, and 185 (12.47%) because they had cervical intraepithelial neoplasia. Therefore, a total of 1,101 women qualified for the study. Twenty-one (1.42%) medical records were not used because they had missing information of the patients due to the non-accomplishment of the oncologic treatment at Inca, and 76 (5.12%) lacked information about the histological type. Thus, a total of 1,004 patients remained in the study (Figure 1).

The process of searching and extracting data from the participants of the original study is described by Silva^13^. Here, we studied sociodemographic variables (age at cancer diagnosis, skin color, level of education, and marital status), reproductive variables (menarche and ages of first sexual activity, pregnancies, abortions, number of children, and menopausal state), clinic variables (comorbidity, tumor staging, and histological type of tumor), and variables related to life habits (alcoholism, smoking, and use of oral contraceptives). The variables that met the completeness criteria defined by Romero and Cunha^14^ were selected for the analysis. Incompleteness refers to the blank fields (incomplete information) of each variable. Completeness is classified as: excellent (variable with less than 5% incomplete filling), good (5% to 10%), regular (10% to 20%), bad (20% to 50%), and very bad (50% or more)^14^.

The variables with good to excellent completeness were analyzed: histological type of the tumor (squamous cell carcinoma and adenocarcinoma), age in years (< 40 and ≥ 40), skin color (white and non-white), education in complete years of study (< 8, 8 to 11 and ≥ 12), marital status (with and without partner), age of menarche in years (< 12 and ≥ 12), age of the first sexual activity in years (≤ 16, 17 to 19 and ≥ 20), pregnancies (yes and no), number of pregnancies (none, 1 and > 2), abortions (yes and no), number of abortions (none, 1 and ≥ 2), number of children (none, 1 to 2 and > 2), comorbidity (yes and no), tumor staging (IA-IIA, IIB-IIIA and IIIB-IV), smoking (never smoked and former smoker or smoker), and alcoholism (has never drunk and former alcoholic or alcoholic). Other variables with regular, bad or very bad completeness were excluded from the analysis: use of oral contraceptives (28.7%, bad), age of first pregnancy (40%, bad), number of natural births (56%, very bad), number of caesarean births (56%, very bad), number of sexual partners (36%, bad), and menopause (22%, bad).

A descriptive analysis of the continuous variables (age; education; ages of menarche, first sexual activity and first pregnancy; number of pregnancies and children), which were categorized using cut-off points based on the measure distribution of central (mean and median) and dispersion (standard deviation and interquartile range) tendencies, besides the cut-off points used in other studies. For the age variable, the cutoff point of 40 years old was chosen to ensure that no women in the younger age range had reached menopause, besides following other studies that also used the cutoff point of < 40 years for women with cervical cancer^2,15^.

For categorical variables, (absolute and relative) frequency distribution was performed. Differences between the distributions of sociodemographic, reproductive, clinical and lifestyle characteristics, according to age and histological type, were evaluated using Fisher’s chi-square and exact tests, with a 5% level of significance.

The odds ratios (OR) of the exposure among women with AC compared with women with SCC were estimated with 95% confidence intervals, using the logistic regression method. The statistical significance was assessed by the Wald test. The variables that presented a significance level of 0.20 in the univariate analysis were eligible for inclusion in the multivariate analysis. Hence, the final model was built both based on the significance level of the variable and on the plausibility of its relationship with the outcome. To evaluate the adjustment of the final model, the residue analysis was executed. All analyses were performed using the SPSS statistical package version 21.0.

## RESULTS

Among the 1,004 women studied, SCC was the most frequent histological type (83.9% of cases). The average age at AC diagnosis was 48.9 years old (SD = 14.076 years) and SCC was 49.5 years old (SD = 14.084 years). Approximately 70% of the women received the diagnosis aged more than 40 years in both histological types, with no statistically significant difference in this distribution. The study mostly included non-white women (67.4%), with less than 8 years of education (51.9%), with onset of sexual activity up to 16 years of age (40.7%), pregnancy history (95.5%), with more than one pregnancy (82.9%) and more than two children (52.7%). Herein, 45.8% of women with cervical cancer were smokers or former smokers (Table 1).

**Table 1:** Distribution of epidemiological and clinical characteristics in the cohort of women diagnosed with cervical cancer in Rio de Janeiro, according to the histological type.

Characteristic	Total^a^	AC	SCC	Test X^2^
			
	n (%)	n (%)	n (%)	p^b^
Total	1,004 (100)	162 (16.1)	842 (83.9)	
Age (years)				1.00
< 40	273 (27.2)	44 (27.2)	229 (27.2)	
≥ 40	731 (72.8)	118 (72.8)	613 (72.8)	
Skin color				0.927
White	327 (32.6)	52 (32.1)	275 (32.7)	
Non-white	677 (67.4)	110 (67.9)	567 in (67.3)	
Education level				**0.002**
≥ 12 years of education	39 (3.9)	12 (7.4)	27 (3.2)	
8 to 11 years of education	444 (44.2)	83 (51.2)	361 (42.9)	
< 8 years of education	521 (51.9)	67 (41.4)	454 (53.9)	
Marital status				0.668
With a partner	495 (49.3)	77 (47.5)	418 (49.6)	
Without a partner	509 (50.7)	85 (52.5)	424 (50.4)	
Menarche				0.918
≥ 12 years old	700 (75.6)	116 (76.3)	584 (75.5)	
<12 years old	226 (24.4)	36 (23.7)	190 (24.5)	
Age of the first sexual intercourse				**0.002**
≥ 20 years old	166 (20.4)	< 40 (28.6)	126 (18.7)	
17 to 19 years old	317 (38.9)	60 (42.9)	257 (38.1)	
≤ 16 years old	332 (40.7)	< 40 (28.6)	292 (43.3)	
Pregnancy history				**< 0.001**
No	44 (4.5)	17 (10.6)	27 (3.3)	
Yes	934 (95.5)	143 (89.4)	791 (96.7)	
Age of first pregnancy				**0.007**
≥ 20 years old	118 (20.8)	9 (10.6)	109 (22.6)	
17 to 19 years old	196 (34.5)	26 (30.6)	170 (35.2)	
≤ 16 years old	254 (44.7)	50 (58.8)	204 (42.2)	
None	44 (4.5)	17 (10.6)	27 (3.3)	
Number of pregnancies				**< 0.001**
1	122 (12.6)	33 (20.6)	89 (11)	
> 1	806 (82.9)	110 (68.8)	696 (85.7)	
Abortions				0.390
No	517 (60.3)	86 (63.7)	431 (59.6)	
Yes	341 (39.7)	49 (36.3)	292 (40.4)	
Number of abortions				0.506
None	525 (60.1)	87 (63.5)	438 (59.4)	
1	213 (24.4)	33 (24.1)	180 (24.4)	
> 1	136 (15.6)	17 (12.4)	119 (16.1)	
Number of children^c^				**< 0.001**
None	20 (2.3)	7 (5.1)	13 (1.7)	
1 to 2	397 (45.1)	78 (56.9)	319 (42.9)	
> 2	464 (52.7)	52 (38.0)	412 (55.4)	
Comorbidities				0.858
No	529 (56)	86 (57)	441 (56)	
Yes	415 (44)	65 (43)	346 (44)	
Smoking				**0.012**
Never smoked	539 (54.2)	102 (63.4)	437 (52.4)	
Smoker or former smoker	456 (45.8)	59 (36.6)	397 (47.6)	
Alcoholism				0.142
Never drank	490 (49.9)	88 (55.3)	402 (48.9)	
Alcoholic or former alcoholic	491 (50.1)	71 (44.7)	420 (51.1)	
Staging				**< 0.001**
IA–IIA	264 (26.5)	63 (39.4)	201 (24.0)	
IIB–IIIA	267 (26.8)	44 (27.5)	223 (26.6)	
IIIB–IV	497 (46.8)	53 (33.1)	414 (49.4)	

AC: Cervical adenocarcinoma; SCC: Squamous cell carcinoma

^a^ Totals can change due to missing data.

^b^ p-value of Fisher’s chi-square or exact tests. Statistically significant values (p < 0.05) are shown in bold.

^c^ Only in women who have been pregnant.

Compared with women diagnosed with SCC, women with AC presented a higher frequency of ≥ 12 years of education (7.4 *% versus* 3.2%), age of first sexual activity ≥ 20 years (28.6% *versus* 18.7%), nulliparity (10.6% *versus* 3.3%), age of first pregnancy ≤ 16 years (58.8% *versus* 42.2%), no children (5.1% *versus* 1.7%), staging IA to IIA Diagnosis (39.4% *versus* 24.0%) and lower smoking frequency (36.6% *versus* 47.6%) (Table 1).

Table 2 shows that among women with initial staging (I-IIA), those with AC presented higher education frequency ≥ 12 years of study (14.3% *versus* 7.5%), absence of a partner (54.0% *versus* 38.3%) and more than two children (48.6% *versus* 30.0%) compared with the ones with histological type SCC. Regarding women in the IIB-IIIA staging, smoking was less frequent among those with AC than among those with SCC (38.6 *versus* 54.3%). An even higher frequency of nulliparity was observed among women with AC when compared with those with SCC, both in the IA-IIA staging (16.1% *versus* 5.1%) and in the IIB-IIIA staging (11.4% *versus* 2.3%).

**Table 2:** Distribution of epidemiological and clinical characteristics in the cohort of women diagnosed with cervical cancer in Rio de Janeiro, according to the histological type.

Characteristic	Stage I-IIA		Stage IIB-IIIA		Stage IIIB-IV	
		
	AC	SCC	AC	SCC	AC	SCC
					
	n (%)	n (%)	n (%)	n (%)	n (%)	n (%)
Total	65 (23.3)	214 (76.7)	44 (15.8)	234 (84.2)	35 (10.9)	286 (89.1)
Age (years)						
< 40	29 (46.0)	87 (43.3)	7 (15.9)	51 (22.9)	8 (15.1)	91 (22.0)
≥ 40	34 (54.0)	114 (56.7)	37 (84.1)	172 (77.1)	45 (84.9)	323 (78.0)
Skin color						
White	24 (38.1)	76 in (37.8)	12 (27.3)	77 (34.5)	16 (30.2)	121 (29.2)
Non-white	39 (61.9)	125 (62.2)	32 (72.7)	146 (65.5)	37 (69.8)	293 (70.8)
Education level						
≥ 12 years of education	**9 (14.3)**	**15 (7.5)**	2 (4.5)	6 (2.7)	1 (1.9)	6 (1.4)
8 to 11 years of education	**37 (58.7)**	**97 (48.3)**	21 (47.7)	82 (36.8)	24 (45.3)	181 (43.7)
< 8 years of education	**17 (27.0)**	**89 (44.3)**	21 (47.7)	135 (60.5)	28 (52.8)	227 (54.8)
Marital status						
With a partner	**29 (46.0)**^b^	**124 (61.7)**^b^	25 (56.8)	101 (45.3)	22 (41.5)	191 (46.1)
Without a partner	**34 (54.0)**^b^	**77 (38.3)**^b^	19 (43.2)	122 (54.7)	31 (58.5)	223 (53.9)
Menarche						
≥ 12 years old	47 (81.0)	145 (76.2)	32 (76.2)	168 (78.1)	36 (72.0)	268 (73.0)
<12 years old	11 (19.0)	44 (23.3)	10 (23.8)	47 (21.9)	14 (28.0)	99 (27.0)
Age of first sexual intercourse						
≥ 20	10 (18.2)	28 (16.8)	**17 (27.7)**^b^	**38 (19.6)**^b^	13 (27.7)	60 (19.3)
17 to 19	27 (49.1)	61 (36.5)	**14 (38.3)**^b^	**71 (36.6)**^b^	18 (38.3)	124 (39.9)
≥ 16	18 (32.7)	78 (46.7)	**6 (34.0)**^b^	**85 (43.8)**^b^	16 (34.0)	127 (40.8)
Pregnancy history						
No	**10 (16.1)**^b^	**10 (5.1)**^b^	**5 (11.4)**^b^	**5 (2.3)**^b^	1 (1.9)	9 (3.0)
Yes	**52 (83.9)**^b^	**188 (94.9)**^b^	**39 (88.6)**^b^	**217 (97.7)**^b^	51 (98.1)	383 (97.0)
Number of pregnancies						
None	**10 (16.1)**^b^	**10 (5.1)**^b^	**5 (11.4)**^b^	**5 (2.3)**^b^	1 (1.9)	12 (3.1)
1	**17 (27.4)**^b^	**22 (11.2)**^b^	**10 (22.7)**^b^	**22 (10)**^b^	6 (11.5)	45 (11.5)
> 1	**35 (56.5)**^b^	**165 (83.8)**^b^	**29 (65.9)**^b^	**193 (87.7)**^b^	45 (86.5)	335 (85.5)
Abortions						
No	31 (64.6)	93 (54.4)	22 (57.9)	121 (61.1)	32 (66.7)	214 (61)
Yes	**17 (35.4)**	78 (45.6)	16 (42.1)	77 (38.9)	16 (33.3)	137 (39)
Number of abortions						
None	32 (64)	94 (54.3)	22 (57.9)	123 (60.3)	32 (66.7)	218 (61.1)
1	10 (20)	51 (29.5)	13 (34.2)	50 (24.5)	10 (20.8)	79 (22.1)
> 1	8 (16)	28 (16.2)	3 (7.9)	31 (15.2)	6 (12.5)	60 (16.8)
Number of children^a^						
None	**3 (1.7)**^b^	**4 (8.0)**^b^	**5 (2.4)**^b^	**2 (5.3)**^b^	5 (1.4)	1 (2.1)
1 to 2	**87 (49.7)**^b^	**31 (62.0)**^b^	**77 (37.2)**^b^	**21 (55.3)**^b^	154 (42.9)	25 (52.1)
> 2	**85 (48.6)**^b^	**15 (30.0)**^b^	**125 (60,4)**^b^	**15 (39.5)**^b^	200 (55.7)	22 (45.8)
Comorbidities						
No	41 (65.1)	119 (59.2)	21 (47.7)	112 (50.2)	24 (45.3)	50.5
Yes	19 (30.2)	70 (34.8)	19 (43.2)	102 (45.7)	25 (47.2)	171 (41.3)
Smoking						
Never smoked	45 (71.4)	122 (60.7)	**27 (61.4)**^b^	**102 (45.7)**^b^	28 (53.8)	211 (52.0)
Smoker or former smoker	18 (28.6)	79 (39.3)	**17 (38.6)**^b^	**121 (54.3)**^b^	24 (46.2)	195 (48.0)
Alcoholism						
Never drank	34 (54.8)	93 (46.2)	25 (58.1)	206 (51.6)	29 (55.8)	164 (57.5)
Alcoholic or former alcoholic	28 (45.2)	107 (53.5)	18 (41.9)	193 (48.4)	23 (44.2)	121 (42.5)

AC: Cervical adenocarcinoma; SCC: Squamous cell carcinoma

^a^ Only in women who have been pregnant.

^b^ p-value of Fisher’s chi-square or exact tests and statistically significant values (p < 0.05) in bold.

In Table 3, in comparison with women with SCC, women with AC had 3.01 (95%CI 1.46–6,23) and 1.56 (95%CI 1.10–2.21) times the chance of having education of ≥ 12 years and 8 to 11 years of study, respectively; they had 2.32 (95%CI 1.43–3,77) and 1.70 (95%CI 1.10–2,63) times the chance of having the first sexual activity at ≥ 20 and 17 to 19 years old, respectively; they had 3.48 (95%CI 1.85–6,55) times the chance of being nulliparous, and 3.98 (95%CI 2.10–7,55) and 2.35 (95%CI 1.50–3,67) times the chance of nulliparity and one pregnancy, respectively; 4.27 (95%CI 1,63–11,18) and 1.94 (95%CI 1.32–2.83) times the chance of having no child and one or two children, respectively; 1.57 (95%CI 1.11–2,22) times the chance of never having smoked and 2.45 (95%CI 1,64–3,66) and 1.54 (95%CI 1.00–2,37) times the chance of being diagnosed in IA-IIA and IIB-IIIA staging, respectively.

**Table 3:** Gross odds ratio of the epidemiological and clinical characteristics in the cohort of women diagnosed with cervical cancer in Rio de Janeiro, according to the histological type.

Characteristic	CA	SCC	Test X^2^	OR (95%CI)
		
	n (%)	n (%)	p^b^	
Total	162 (16.1)	842 (83.9)		
Age (years)			1.00	
< 40	44 (16.1)	229 (83.9)		0.10 (0.68–1.46)
≥ 40	118 (16.1)	613 (83.9)		1
Skin color			0.927	
White	52 (15.9)	275 (84.1)		1
Non-white	110 (16.2)	567 (83.8)		1.03 (0.72–1.47)
Education level			**0.002**	
≥ 12 years of education	12 (30.8)	27 (69.2)		**3.01 (1.46–6.23)**
8 to 11 years of education	83 (18.7)	361 (81.3)		**1.56 (1.10–2.21)**
< 8 years of education	67 (12.9)	454 (87.1)		1
Marital status			0.668	
With a partner	77 (15.6)	418 (84.4)		1
Without a partner	85 (16.7)	424 (83.3)		1.09 (0.78–1.52)
Menarche			0.918	
≥ 12 years old	116 (16.6)	584 (83.4)		1.05 (0.70–1.58)
<12 years old	36 (15.9)	190 (84.1)		1
Age of the first sexual intercourse			**0.002**	
≥ 20 years old	40 (24.1)	126 (75.9)		**2.32 (1.43–3.77)**
17 to 19 years old	60 (18.9)	257 (81.1)		**1.70 (1.10–2.63)**
≤ 16 years old	40 (12.0)	292 (88.0)		1
Pregnancy history			**< 0.001**	
No	17 (38.6)	27 (61.4)		**3.48 (1.85–6.55)**
Yes	143 (15.3)	791 (84.7)		1
Number of pregnancies			**< 0.001**	
None	17 (38.6)	27 (61.4)		3.98
1	33 (27)	89 (73)		**2.35 (1.50–3.67)**
> 1	110 (13.6)	696 (86.4)		1
Abortions			0.390	
No	86 (16.6)	431 (83.4)		1
Yes	49 (14.4)	292 (85.6)		0.84 (0.57–1.23)
Number of abortions			**0.506**	
None	87 (16.6)	438 (83.4)		1.40 (0.80–2.43)
1	33 (15.5)	180 (84.5)		1.28 (0.68–2.41)
> 1	17 (12.5)	119 (87.5)		1
Number of children^a^				
None	7 (35.0)	13 (65.0)	**< 0.001**	**4.27 (1.63–11.18)**
1 to 2 children	78 (19.6)	319 (80.4)		**1.94 (1.32–2.83)**
> 2 Children	52 (11.2)	412 (88.8)		1
Comorbidities			0.858	
No	86 (16.3)	441 (83.7)		1
Yes	65 (15.8)	346 (84.2)		0.96 (0.68–1.37)
Smoking				
Never smoked	102 (18.9)	437 (81.1)	**0.012**	**1.57 (1.11–2.22)**
Smoker or former smoker	59 (12.9)	397 (87.1)		1
Alcoholism			0.142	
Never drank	88 (18.0)	402 (82.0)		1
Alcoholic or former alcoholic	71 (14.5)	420 (85.5)		0.78 (0.55–1.09)
Stage			**< 0.001**	
IA-IIA	63 (23.9)	201 (76.1)		**2.45 (1.64–3.66)**
IIB-IIIA	44 (16.5)	223 (83.5)		**1.54 (1.00–2.37)**
IIIB-IV	53 (11.3)	414 (88.7)		1

AC: Cervical adenocarcinoma; SCC: Squamous cell carcinoma

^a^ Only in women who have been pregnant.

^b^ p-value of Fisher’s chi-square or exact tests. Statistically significant values (p < 0.05) in bold.

^c^ Gross odds ratio

In the multiple logistic regression analysis, we observed that the staging IA-IIA was positively associated (OR = 1.79; 95%CI 1.03–3.13) with the AC, regardless of age, education, age of first sexual activity and number of children. Similarly, education greater than or equal to 12 years of study (OR = 3.34; 95%CI 1.27–8,76), no children (OR = 3.5; 95%CI 1.27–9,85) or up to two children (OR = 1.6; 95%CI 1.02–2.50) also remained positively associated with the AC, regardless of age and other variables of the model (Table 4).

**Table 4:** Adjusted odds ratio and respective 95% confidence interval estimated for the cohort of women with cervical cancer, according to the histological type.

Characteristic	Adjusted OR*	95%CI
Stage		
IA-IIA	**1.79**	**(1.03-3.13)**
IIB-IIIA	1.00	(0.56–1.75)
IIIB-IV	1	
Education level		
< 8 years of education	1	
8 to 11 years of education	1.27	(0.77–2.10)
≥ 12 years of education	**6.30**	**(1.97–20.13)**
Age of the first sexual intercourse		
≤ 16 years old	1	
17 to 19 years old	1.38	(0.82–2.34)
≥ 20 years old	1.57	(0.82–3.03)
Number of children		
None	**3.81**	**(1.20–12.08)**
1 to 2	**1.74**	**(1.05–2.87)**
> 2	1	

-2Log-Likelihood: 534.805*

* Odds ratios adjusted for age (continuous) and other variables of the model.

Statistically significant values in bold.

## DISCUSSION

Results show that the most frequent histological type was SCC (83.9%), compared with AC (16.1%), corroborating other national studies in which the frequency of AC ranged from 9.7% (hospital study conducted in Porto Alegre from 2005 to 2006^16^) and 12% (study conducted at INCA, Rio de Janeiro, from 1999 to 2004^11^). Other hospital-based studies estimated a frequency that ranged from 14% in South Korea (1988 – 2008)^17^to 20% in Japan (2001 – 2010)^18^. Higher indices only come from developed countries and can be attributed to a better control of cervical cancer of the squamous type in these areas. Over the last decades, in the United States and other developed countries, the incidence of SCC reduced steadily, while the incidence of AC and its variants in relative and absolute terms increased, due to the rise in detection of precursor lesions of SCC from the organization of screening programs. That differs from the AC, because, as studies suggest, cytology-based screening is more effective for detecting the precursor lesions of SCC.

Mean age at diagnosis according to histological type presented no statistical significance in the studied period. These findings are consistent with some studies that found a mean age varied from 47 years (26 – 69 years old) in Italy between 2003 and 2010^19^, and 53 years (SD = 12 years) in Japan between 2001 and 2010^18^ for the AC and; while for SCC, the mean age was 47 years (22 – 73 years) in Japan between 1984 and 2003^20^, and 51 years old (SD = 13) in another study conducted in the same country between 2001 and 2010^18^.

Moreover, the main finding of this study was the positive association between AC and the earlier staging (IA-IIA), suggesting that women with AC were more likely to be diagnosed earlier than women with SCC. Similar results were found in a retrospective population-based study conducted in the USA (1988 – 2005) with 24,562 women diagnosed with stages IB1 to IVB of cervical cancer. The authors observed that patients with AC had a higher frequency of the disease (26%) at the initial stage (IB) at the time of diagnosis in comparison with 16.9% of women with SCC at the same stage, reinforcing that these histological types are distinct clinical entities in their form of presentation^21^. Similarly, a retrospective cohort study conducted in the USA (1973 – 2002) found that the frequency of women diagnosed with AC at stage IB was also higher than in the SCC histological type for the same stage (71% *versus* 51%, respectively)^3^. Likewise, a study conducted in a Brazilian hospital between 2000 and 2009 using information from 239 hospitals found that stage I was more frequent in women diagnosed with AC (33.3%), while 21.3% of women presented SCC^12^. This shows that the profile of these women is different and reflects the issues of access to health services and level of education.

Corroborating these findings, our results also indicated that women with AC exhibited a higher level of education than women with SCC. Moreover, the analysis stratified by histological type and staging revealed that women with AC and stage I-IIA had a higher level of education (14.3%) compared with those with SCC (7.5%) (Table 2). Evidence suggests a direct association between education level, socioeconomic status and health services accessibility. Low level of education, which is usually used as a substitute variable of socioeconomic status is associated with the risk for cervical cancer, suggesting that these women may neglect the importance of the exam or lack the necessary knowledge to seek screening and treatment, reflecting the absence of health services accessibility^22^. These findings corroborate Carmo and Luiz^11^ retrospective hospital-based study conducted at INCA (2003 – 2010) that observed that women with 11 years or more of education had a higher frequency of stages I and II of the diagnosis of cervical cancer, and those with the histological type SCC were more commonly diagnosed at more advanced stages of the International Federation of Gynecology and Obstetrics – FIGO (p < 0.001); while women with AC were more frequently diagnosed at an early stage. Women with higher education level generally seek health care and have more knowledge about prevention, that way their disease is detected at an early stage, promoting a more successful treatment^11^, which explains the association.

This study supports a positive association between nulliparity and AC. This finding is consistent with the results of a hospital-based study developed between 1992 and 1996 with an American population of women with SCC and AC^9^ The authors observed a negative association between those who gestated and the AC (OR = 0.4; 95%CI 0.2–0.8) and positive association between five or more pregnancies and SCC (OR = 2.2; 95%CI 0.9–5.4). These findings suggest that reproductive events, especially those related to endogenous exposure to sex hormones, may have different effects on the progression of HPV infection, affecting the histological type of cervical cancer^9^. Studies show that, in addition to HPV infection, which is the necessary cause for cervical cancer, the histological types AC and SCC share other risk factors, such as the number of sexual partners, age of the first sexual activity, age of first pregnancy, and use of oral contraceptives^4,8,9,23^. Smoking and multiparity are risk factors directly associated with SCC and inversely associated with AC^9,10^. However, some authors maintain that the AC may represent a histological entity that shares the risk factors related to endometrial cancer, such as obesity, the use of oral contraceptives and nulliparity, in addition to the inverse association with smoking^24^. Nulliparous women have a higher number of ovulatory menstrual cycles due to the absence of pregnancy and lactation, with higher cumulative exposure to estrogen hormone and/or lower exposure to progesterone hormone^24^ Progesterone directly affects cancer cells, inhibiting neoplastic cell growth and cell invasion^25^

We also observed that women with AC initiated sexual activity more frequently between 17 and 19 years of age, while women with SCC initiated sexual activity earlier (≤ 16 years). Similarly, in the hospital-based study that Altekruse et al.^9^ developed with the American population, they observed that early age in the first sexual activity (< 17 years old *versus* ≥ 20 years old of the reference group) was associated with SCC (OR = 2.0; 95%CI 1.0–3.9), but not with AC (OR = 0.9; 95%CI 0.5–1.8). This suggests that other endogenous and environmental factors, different from those known for SCC, could act as cofactors modulating the risk of AC^9^. Our findings suggest that factors related to exposure to endogenous estrogen, such as nulliparity and having up to two children could comprise this group of cofactors.

This study also observed a lower frequency of smoking in women with AC, with no association among AC and smoking. Castellsagué et al.^4^ observed similar findings with a joint analysis of data of case-control studies of AC conducted and coordinated by IARC in eight countries (Algeria, Morocco, Brazil, Peru, Paraguay, India, Thailand and the Philippines) between 1985 and 1997. The authors did not observe statistically significant associations between smoking and AC, although they observed a positive association between smoking and SCC^4^. Some authors^8,9^ reported smoking as a risk factor for SCC, but not for AC. This finding is consistent with the hypothesis that glandular cells are exposed to carcinogens during periods of increased ectopia, which is lower in women who smoke^26^. In addition, nicotine was reported to stimulate the growth of epithelial cells in healthy women^27^ and the growth of ectocervical cells immortalized by HPV^28^. Thus, the lack of exposure to carcinogens of cigarette smoke due to decreased ectopia and increased growth of ectocervical cells by nicotine could be responsible for the absence of association between smoking and AC^23^.

This study has limitations inherent to retrospective studies based on medical records, concerning to missing data. Among the limitations of this investigation, one is the absence of further investigation of some important exposures associated with AC presented in other studies, such as the use of oral contraceptives^9,23,29^, number of sexual partners and menopause age^30^. Moreover, this is a hospital-based cohort, not representing what occurs in the population-based cohort of Rio de Janeiro. However, this study was the first one in Brazil that evaluated the association of exposure factors between the histological types AC and SCC, with the major sample size of AC histological type (n = 162), and with more accurate data from a search not only in the HBCR, but also directly in the medical records.

## CONCLUSION

The frequency of AC observed in this study (16.1%) was similar to those observed elsewhere in Brazil and the world. In comparison with women with SCC, women with AC had higher education level, later sexual initiation, nulliparity or maximum of two children, lower smoking frequency, and predominance of cancer diagnosis with earlier staging. The present findings highlight the difference between the histological types, suggesting that AC may represent a distinct clinical entity of cervical neoplasia, which may require different approaches to SCC. Studies with different epidemiological designs and larger sample size are needed to test the hypotheses raised related to the associations between the exposure factors analyzed in the present study and the different histological types of cervical cancer.
