# Supplementary material for: Characterization of women with cervical cancer assisted at Inca by histological type
Source: Rev Saude Publica. 2019 Sep 27;53:88. doi: 10.11606/s1518-8787.2019053001218 (PMC6776112; doi:10.11606/s1518-8787.2019053001218)
Supplement: Translation

# Translation: Caracterização de mulheres com câncer cervical atendidas no Inca por tipo histológico

**Authors:** Suelem do Rozario, Iléia Ferreira da Silva, Rosalina Jorge Koifman, Ilce Ferreira da Silva

**Affiliations:** I Brasil Fundação Oswaldo Cruz. Escola Nacional de Saúde Pública Sérgio Auroca. Programa de Saúde Pública e Meio Ambiente. Rio de Janeiro, RJ, Brasil; II Brasil Fundação Oswaldo Cruz. Escola Nacional de Saúde Pública Sérgio Auroca. Departamento de Epidemiologia e Métodos Quantitativos em Saúde. Rio de Janeiro, RJ, Brasil

**Keywords:** Neoplasias do Colo do Útero, epidemiologia, Teste de Papanicolaou, classificação, História Reprodutiva, Fatores de Risco, Fatores Socioeconômicos

## Abstract

**OBJETIVO:**

Determinar a distribuição das características sociodemográficas, reprodutivas, clínicas e de hábitos de vida na coorte de mulheres diagnosticadas com câncer cervical, atendidas no Inca entre 2012 e 2014, segundo o tipo histológico.

**MÉTODOS:**

Estudo observacional retrospectivo de uma coorte hospitalar de 1.004 mulheres diagnosticadas com câncer cervical. Os dados foram obtidos pelo Registro Hospitalar de Câncer do Inca, prontuários físicos e eletrônicos.

**RESULTADOS:**

O tipo histológico mais frequente foi o carcinoma de células escamosas (83,9%). Aproximadamente 70% das mulheres foram diagnosticadas com mais de 40 anos de idade. Houve a predominância de mulheres não brancas (67,4%), com menos de 8 anos de escolaridade (51,9%), com início da atividade sexual até 16 anos de idade (40,7%), que já engravidaram alguma vez na vida (95,5%), com mais de uma gestação (82,9%) e mais de dois filhos (52,7%); 45,8% das mulheres eram tabagistas ou ex-tabagistas. O adenocarcinoma cervical esteve positivamente associado ao estadiamento mais precoce (IA-IIA) (OR = 1,79; IC95% 1,03–3,13), assim como a mulheres com ≥ 12 anos de estudo (OR = 6,30; IC95% 1,97–20,13), que não tiveram filhos (OR = 3,81; IC95% 1,20–12,08) ou que tiveram até dois filhos (OR = 1,74; IC95% 1,05–2,87).

**CONCLUSÕES:**

Destaca-se a diferença entre os tipos histológicos, sugerindo que as mulheres com adenocarcinoma cervical possam representar uma entidade clínica distinta de neoplasia cervical, podendo demandar abordagens diferentes das utilizadas no carcinoma de células escamosas.

## INTRODUÇÃO

O câncer cervical é uma das principais causas de morte por câncer na população feminina no mundo, especialmente nas regiões menos desenvolvidas, onde ocorrem 83% dos casos novos e 86% dos óbitos. Segundo estimativas da Agência Internacional de Pesquisa em Câncer (IARC), em 2012 as taxas de incidência e mortalidade da doença no Brasil foram 14/100.000 e 6,8/100.000 habitantes, respectivamente^1^.

A implementação de programas de rastreamento e tratamento das lesões precursoras do câncer cervical tem levado à queda tanto das taxas de incidência quanto de mortalidade do câncer cervical de células escamosas nos últimos 50 anos nos países desenvolvidos^2,3^ e, nas décadas mais recentes, também em alguns países em desenvolvimento^4,5^. Essa neoplasia pode apresentar-se com diferentes tipos histológicos, dos quais o carcinoma de células escamosas (SCC) é o mais frequente (80%), enquanto o adenocarcinoma cervical (AC) e o carcinoma adenoescamoso (ASC) representam 10-15% dos casos^6^. Entretanto, tem sido observado um aumento da incidência do AC nos países desenvolvidos, especialmente em mulheres com idade entre 20 e 40 anos^2^.

A infecção pelo papilomavírus humano (HPV) é a causa necessária, mas não suficiente do câncer de colo uterino, sendo requerida a exposição a co-fatores para que o fenótipo tumoral ocorra^4,7^. Entretanto, alguns cofatores de risco estão especificamente associados ao AC. Estudos destacam que, além da imunossupressão^8^ e dos fatores ligados ao comportamento sexual^9^, que são comuns a ambos os tipos histológicos, o uso de contraceptivo oral (CO)^4,9^, a obesidade^10^ e a nuliparidade^9^ parecem estar particularmente relacionados a esse subtipo.

A baixa frequência dessa neoplasia nos países desenvolvidos, especialmente do adenocarcinoma cervical, tem limitado a avaliação dos fatores associados. Assim, os países em desenvolvimento, que apresentam alta frequência desse câncer, teriam uma oportunidade de análise mais robusta em função da possibilidade de maior casuística. Apesar disso, além de existirem poucos estudos no Brasil que avaliaram o perfil da distribuição dos fatores associados ao câncer cervical segundo o tipo histológico, os dois maiores foram realizados com base apenas nas informações contidas no Registro Hospitalar de Câncer (RHC), e nenhum deles estimou quais fatores estavam associados ao tipo histológico^11,12^. Dessa forma, o objetivo deste estudo foi determinar a distribuição das características sociodemográficas, reprodutivas, clínicas e de hábitos de vida na coorte de mulheres diagnosticadas com câncer cervical, atendidas no Inca entre 2012 e 2014, segundo o tipo histológico.

## MÉTODOS

O presente estudo é um subprojeto da pesquisa intitulada *“*Avaliação do tempo de espera no manejo terapêutico e seus efeitos na sobrevida de mulheres diagnosticadas com câncer de colo de útero em uma coorte hospitalar no INCA II”, aprovado pelo Comitê de Ética em Pesquisa do Instituto Nacional de Câncer José Alencar Gomes da Silva (Inca) em outubro de 2015.

Foi realizado um estudo observacional descritivo e exploratório da coorte de mulheres com diagnóstico primário de câncer cervical matriculadas e tratadas num centro que concentra todos os casos de câncer ginecológico do Inca, entre julho de 2012 e outubro de 2014.

A população do estudo foi composta pelo universo dos casos de câncer cervical primário – codificado como C53.0 pela 10^a^ Revisão da Classificação Internacional de Doenças (CID10) – com confirmação histopatológica dos seguintes tipos histológicos: escamoso, adenoescamoso e adenocarcinoma. Os critérios de exclusão foram: casos de carcinoma de colo uterino *in situ* e ter iniciado o tratamento fora do Inca.

Com base na identificação dos casos classificados no RHC, foi efetuada a revisão dos prontuários físicos e eletrônicos e dos laudos histopatológicos. No período de julho de 2012 a outubro de 2014, foram matriculadas 1.483 mulheres com câncer cervical. Dessas, 242 (16,31%) foram excluídas por apresentarem erro de classificação do tipo histológico, 140 (9,44%) por terem sido encaminhadas de outra instituição de saúde apenas para a realização de braquiterapia e 185 (12,47%) por terem neoplasia intraepitelial cervical. Assim, um total de 1.101 mulheres foram elegíveis para o estudo. Dessas, 21 (1,42%) prontuários foram considerados perdas por não conterem as informações das pacientes devido à não realização do tratamento oncológico no Inca e 76 (5,12%) por não conterem informações sobre o tipo histológico. Desse modo, permaneceram no estudo um total de 1.004 pacientes (Figura 1).

**Figura 1:** Fluxo da seleção de mulheres para o estudo.

O processo de busca e extração dos dados das participantes do estudo original está descrito em Silva^13^. No presente estudo, foram estudadas as variáveis sociodemográficas (idade ao diagnóstico de câncer, cor da pele, escolaridade e estado conjugal), reprodutivas (idades da menarca e da sexarca, gestações, abortos, número de filhos e estado menopausal), clínicas (comorbidade, estadiamento tumoral e tipo histológico do tumor) e relacionadas aos hábitos de vida (etilismo, tabagismo e uso de anticoncepcional oral). As variáveis que cumpriram os critérios de completitude definidos por Romero e Cunh^14^ foram selecionadas para a análise. A incompletude refere-se aos campos em branco (informação incompleta) de cada variável. A completitude é classificada em: excelente (variável com menos de 5% de preenchimento incompleto), bom (5% a 10%), regular (10% a 20%), ruim (20% a 50%) e muito ruim (50% ou mais)^14^.

Foram analisadas as variáveis com completitude boa a excelente: tipo histológico do tumor (carcinoma de células escamosas e adenocarcinoma), idade em anos (< 40 e ≥ 40), cor da pele (branca e não branca), escolaridade em anos completos de estudo (< 8, de 8 a 11 e ≥ 12), estado conjugal (com e sem companheiro), idade da menarca em anos (< 12 e ≥ 12), idade da sexarca em anos (≤ 16, de 17 a 19 e ≥ 20), gestações (sim e não), número de gestações (nenhuma, 1 e > 2), abortos (sim e não), número de abortos (nenhum, 1 e ≥ 2), número de filhos (nenhum, 1 a 2 e > 2), comorbidade (sim e não), estadiamento tumoral (IA-IIA, IIB-IIIA e IIIB-IV), tabagismo (nunca fumou e ex-fumante ou fumante) e alcoolismo (nunca bebeu e ex-alcoolista ou alcoolista). Outras variáveis com completitude regular, ruim ou muito ruim foram excluídas da análise: uso de anticoncepcional oral (28,7%, ruim), idade da primeira gestação (40%, ruim), número de partos normais (56%, muito ruim), número de partos cesáreos (56%, muito ruim), número de parceiros sexuais (36%, ruim) e menopausa (22%, ruim).

Foi realizada uma análise descritiva das variáveis contínuas (idade; escolaridade; idades da menarca, sexarca e da primeira gestação; número de gestações e de filhos), as quais foram categorizadas utilizando pontos de cortes baseados na distribuição das medidas de tendência central (média e mediana) e de dispersão (desvio-padrão e intervalo interquartis), além dos pontos de corte utilizados em outros estudos. Para a variável idade, foi escolhido o ponto de corte de 40 anos para garantir que na faixa mais jovem não houvesse mulheres que já entraram na menopausa, além de seguir outros estudos que também utilizaram o ponto de corte < 40 anos para mulheres com câncer cervical^2,15^.

Para as variáveis categóricas, foi realizada a distribuição de frequências (absoluta e relativa). As diferenças entre as distribuições das características sociodemográficas, reprodutivas, clínicas e relacionadas aos hábitos de vida, segundo a idade e o tipo histológico, foram avaliadas por meio dos testes do qui-quadrado e exato de Fisher, com um grau de significância de 5%.

As razões de chances (OR) da exposição entre as mulheres com AC comparadas àquelas com SCC foram estimadas com intervalos de confiança de 95%, usando o método de regressão logística. A significância estatística foi avaliada pela estatística de Wald. As variáveis que apresentaram um grau de significância de 0,20 na análise univariada foram elegíveis para inclusão na análise multivariada. Dessa forma, o modelo final foi construído tanto com base no grau de significância da variável quanto na plausibilidade da sua relação com o desfecho. Para avaliar o ajuste do modelo final, foi realizada a análise de resíduos. Todas as análises foram feitas utilizando o pacote estatístico SPSS versão 21.0.

## RESULTADOS

Entre as 1.004 mulheres estudadas, o tipo histológico mais frequente foi o SCC (83,9% dos casos). A média de idade ao diagnóstico de AC foi 48,9 anos (DP = 14,076 anos) e de SCC foi 49,5 anos (DP = 14,084 anos). Aproximadamente 70% das mulheres foram diagnosticadas com mais de 40 anos de idade em ambos os tipos histológicos, sem diferença estatisticamente significativa nessa distribuição. Houve uma predominância de mulheres não brancas (67,4%), com menos de 8 anos de estudo (51,9%), com início da atividade sexual até 16 anos de idade (40,7%), histórico de gestação (95,5%), com mais de uma gestação (82,9%) e mais de dois filhos (52,7%). Neste estudo, 45,8% das mulheres com câncer de colo uterino eram tabagistas ou ex-tabagistas (Tabela 1).

**Tabela 1:** Distribuição das características epidemiológicas e clínicas na coorte de mulheres com diagnóstico de câncer cervical no Rio de Janeiro, segundo o tipo histológico.

Variável	Total^a^	AC	SCC	Teste X^2^
			
	n %)	n (%)	n (%)	p^b^
Total	1.004 (100)	162 (16,1)	842 (83,9)	

Idade (anos)				1,00
< 40	273 (27,2)	44 (27,2)	229 (27,2)	
≥ 40	731 (72,8)	118 (72,8)	613 (72,8)	
Cor da pele				0,927
Branca	327 (32,6)	52 (32,1)	275 (32,7)	
Não branca	677 (67,4)	110 (67,9)	567 (67,3)	
Escolaridade				**0,002**
≥ 12 anos de estudo	39 (3,9)	12 (7,4)	27 (3,2)	
8 a 11 anos de estudo	444 (44,2)	83 (51,2)	361 (42,9)	
< 8 anos de estudo	521 (51,9)	67 (41,4)	454 (53,9)	
Estado conjugal				0,668
Com companheiro	495 (49,3)	77 (47,5)	418 (49,6)	
Sem companheiro	509 (50,7)	85 (52,5)	424 (50,4)	
Menarca				0,918
≥ 12 anos	700 (75,6)	116 (76,3)	584 (75,5)	
< 12 anos	226 (24,4)	36 (23,7)	190 (24,5)	
Idade da sexarca				**0,002**
≥ 20 anos	166 (20,4)	40 (28,6)	126 (18,7)	
17 a 19 anos	317 (38,9)	60 (42,9)	257 (38,1)	
≤ 16 anos	332 (40,7)	40 (28,6)	292 (43,3)	
Histórico de gestação				**< 0,001**
Não	44 (4,5)	17 (10,6)	27 (3,3)	
Sim	934 (95,5)	143 (89,4)	791 (96,7)	
Idade da primeira gestação				**0,007**
≥ 20 anos	118 (20,8)	9 (10,6)	109 (22,6)	
17 a 19 anos	196 (34,5)	26 (30,6)	170 (35,2)	
≤ 16 anos	254 (44,7)	50 (58,8)	204 (42,2)	
Nenhuma	44 (4,5)	17 (10,6)	27 (3,3)	
Número de gestações				**< 0,001**
1	122 (12,6)	33 (20,6)	89 (11)	
> 1	806 (82,9)	110 (68,8)	696 (85,7)	
Abortos				0,390
Não	517 (60,3)	86 (63,7)	431 (59,6)	
Sim	341 (39,7)	49 (36,3)	292 (40,4)	
Número de abortos				0,506
Nenhum	525 (60,1)	87 (63,5)	438 (59,4)	
1	213 (24,4)	33 (24,1)	180 (24,4)	
> 1	136 (15,6)	17 (12,4)	119 (16,1)	
Número de filhos^c^				**< 0,001**
Nenhum	20 (2,3)	7 (5,1)	13 (1,7)	
1 a 2	397 (45,1)	78 (56,9)	319 (42,9)	
> 2	464 (52,7)	52 (38,0)	412 (55,4)	
Comorbidades				0,858
Não	529 (56)	86 (57)	441 (56)	
Sim	415 (44)	65 (43)	346 (44)	
Tabagismo				**0,012**
Nunca fumou	539 (54,2)	102 (63,4)	437 (52,4)	
Fumante ou ex-fumante	456 (45,8)	59 (36,6)	397 (47,6)	
Alcoolismo				0,142
Nunca bebeu	490 (49,9)	88 (55,3)	402 (48,9)	
Alcoolista ou ex-alcoolista	491 (50,1)	71 (44,7)	420 (51,1)	
Estadiamento				**< 0,001**
IA–IIA	264 (26,5)	63 (39,4)	201 (24,0)	
IIB–IIIA	267 (26,8)	44 (27,5)	223 (26,6)	
IIIB–IV	497 (46,8)	53 (33,1)	414 (49,4)	

AC: adenocarcinoma cervical; SCC: carcinoma de células escamosas

^a^ Os totais podem mudar devido aos dados faltantes.

^b^ p-valor dos testes do qui-quadrado ou exato de Fisher. Valores estatisticamente significativos (p < 0,05), estando em negrito os valores estatisticamente significativos (p < 0,05).

^c^ Apenas em mulheres que gestaram.

Quando comparadas às mulheres diagnosticadas com SCC, aquelas com AC apresentaram uma maior frequência de ≥ 12 anos de escolaridade (7,4% *versus* 3,2%), idade da sexarca ≥ 20 anos (28,6% *versus* 18,7%), nuliparidade (10,6% *versus* 3,3%), idade da primeira gestação ≤ 16 anos (58,8% *versus* 42,2%), nenhum filho (5,1% *versus* 1,7%), estadiamento IA a IIA ao diagnóstico (39,4% *versus* 24,0%) e menor frequência de tabagismo (36,6% *versus* 47,6%) (Tabela 1).

Na Tabela 2 observou-se que, dentre as mulheres com estadiamento inicial (I-IIA), aquelas com AC apresentaram maior frequência de escolaridade ≥ 12 anos de estudo (14,3% *versus* 7,5%), ausência de companheiro (54,0% *versus* 38,3%) e mais de 2 filhos (48,6% *versus* 30,0%) que as de tipo histológico SCC. Nas mulheres com estadiamento IIB-IIIA, o tabagismo foi menos frequente entre aquelas com AC comparadas àquelas com SCC (38,6 *versus* 54,3%). Observou-se ainda maior frequência de nuliparidade entre as mulheres com AC quando comparadas àquelas com SCC, tanto no estadiamento IA-IIA (16,1% ver*sus* 5,1%) quanto no estadiamento IIB-IIIA (11,4% *versus* 2,3%).

**Tabela 2:** Distribuição das características epidemiológicas e clínicas por tipo histológico na coorte de mulheres com diagnóstico de câncer cervical no Rio de Janeiro, segundo o estadiamento.

Variável	Estádio I-IIA		Estádio IIB-IIIA		Estádio IIIB-IV	
		
	AC	SCC	AC	SCC	AC	SCC
					
	n (%)	n (%)	n (%)	n (%)	n (%)	n (%)
Total	65 (23,3)	214 (76,7)	44 (15,8)	234 (84,2)	35 (10,9)	286 (89,1)

Idade (anos)						
< 40	29 (46,0)	87 (43,3)	7 (15,9)	51 (22,9)	8 (15,1)	91 (22,0)
≥ 40	34 (54,0)	114 (56,7)	37 (84,1)	172 (77,1)	45 (84,9)	323 (78,0)
Cor da pele						
Branca	24 (38,1)	76 (37,8)	12 (27,3)	77 (34,5)	16 (30,2)	121 (29,2)
Não branca	39 (61,9)	125 (62,2)	32 (72,7)	146 (65,5)	37 (69,8)	293 (70,8)
Escolaridade						
≥ 12 anos de estudo	**9 (14,3)**	**15 (7,5)**	2 (4,5)	6 (2,7)	1 (1,9)	6 (1,4)
8 a 11 anos de estudo	**37 (58,7)**	**97 (48,3)**	21 (47,7)	82 (36,8)	24 (45,3)	181 (43,7)
< 8 anos de estudo	**17 (27,0)**	**89 (44,3)**	21 (47,7)	135 (60,5)	28 (52,8)	227 (54,8)
Estado conjugal						
Com companheiro	**29 (46,0)**^b^	**124 (61,7)**^b^	25 (56,8)	101 (45,3)	22 (41,5)	191 (46,1)
Sem companheiro	**34 (54,0)**^b^	**77 (38,3)**^b^	19 (43,2)	122 (54,7)	31 (58,5)	223 (53,9)
Menarca						
≥ 12 anos	47 (81,0)	145 (76,2)	32 (76,2)	168 (78,1)	36 (72,0)	268 (73,0)
< 12 anos	11 (19,0)	44 (23,3)	10 (23,8)	47 (21,9)	14 (28,0)	99 (27,0)
Idade da sexarca (anos)						
≥ 20	10 (18,2)	28 (16,8)	**17 (27,7)**^b^	**38 (19,6)**^b^	13 (27,7)	60 (19,3)
17 a 19	27 (49,1)	61 (36,5)	**14 (38,3)**^b^	**71 (36,6)**^b^	18 (38,3)	124 (39,9)
≥ 16	18 (32,7)	78 (46,7)	**6 (34,0)**^b^	**85 (43,8)**^b^	16 (34,0)	127 (40,8)
Histórico de gestação						
Não	**10 (16,1)**^b^	**10 (5,1)**^b^	**5 (11,4)**^b^	**5 (2,3)**^b^	1 (1,9)	9 (3,0)
Sim	**52 (83,9)**^b^	**188 (94,9)**^b^	**39 (88,6)**^b^	**217 (97,7)**^b^	51 (98,1)	383 (97,0)
Número de gestações						
Nenhuma	**10 (16,1)**^b^	**10 (5,1)**^b^	**5 (11,4)**^b^	**5 (2,3)**^b^	1 (1,9)	12 (3,1)
1	**17 (27,4)**^b^	**22 (11,2)**^b^	**10 (22,7)**^b^	**22 (10)**^b^	6 (11,5)	45 (11,5)
> 1	**35 (56,5)**^b^	**165 (83,8)**^b^	**29 (65,9)**^b^	**193 (87,7)**^b^	45 (86,5)	335 (85,5)
Abortos						
Não	31 (64,6)	93 (54,4)	22 (57,9)	121 (61,1)	32 (66,7)	214 (61)
Sim	17 (35,4)	78 (45,6)	16 (42,1)	77 (38,9)	16 (33,3)	137 (39)
Número de abortos						
Nenhum	32 (64)	94 (54,3)	22 (57,9)	123 (60,3)	32 (66,7)	218 (61,1)
1	10 (20)	51 (29,5)	13 (34,2)	50 (24,5)	10 (20,8)	79 (22,1)
> 1	8 (16)	28 (16,2)	3 (7,9)	31 (15,2)	6 (12,5)	60 (16,8)
Número de filhos^a^						
Nenhum	**3 (1,7)**^b^	**4 (8,0)**^b^	**5 (2,4)**^b^	**2 (5,3)**^b^	5 (1,4)	1 (2,1)
1 a 2	**87 (49,7)**^b^	**31 (62,0)**^b^	**77 (37,2)**^b^	**21 (55,3)**^b^	154 (42,9)	25 (52,1)
> 2	**85 (48,6)**^b^	**15 (30,0)**^b^	**125(60,4)**^b^	**15 (39,5)**^b^	200 (55,7)	22 (45,8)
Comorbidades						
Não	41 (65,1)	119 (59,2)	21 (47,7)	112 (50,2)	24 (45,3)	209 (50,5)
Sim	19 (30,2)	70 (34,8)	19 (43,2)	102 (45,7)	25 (47,2)	171 (41,3)
Tabagismo						
Nunca fumou	45 (71,4)	122 (60,7)	**27 (61,4)**^b^	**102 (45,7)**^b^	28 (53,8)	211 (52,0)
Fumante ou ex-fumante	18 (28,6)	79 (39,3)	**17 (38,6)**^b^	**121 (54,3)**^b^	24 (46,2)	195 (48,0)
Alcoolismo						
Nunca bebeu	34 (54,8)	93 (46,2)	25 (58,1)	206 (51,6)	29 (55,8)	164 (57,5)
Alcoolista ou ex-alcoolista	28 (45,2)	107 (53,5)	18 (41,9)	193 (48,4)	23 (44,2)	121 (42,5)

AC: adenocarcinoma cervical; SCC: carcinoma de células escamosas

^a^ Apenas em mulheres que gestaram.

^b^ p-valor dos testes qui-quadrado ou exato de Fisher e valores estatisticamente significativos (p < 0,05) em negrito.

Na Tabela 3, comparadas com as mulheres com SCC, aquelas com AC tiveram 3,01 (IC95% 1,46–6,23) e 1,56 (IC95% 1,10–2,21) vezes a chance de ter escolaridade ≥ 12 anos de estudo e de oito a 11 anos de estudo, respectivamente; apresentaram 2,32 (IC95% 1,43–3,77) e 1,70 (IC95% 1,10–2,63) vezes a chance de ter a sexarca com ≥ 20 anos e de 17 a 19 anos, respectivamente; tiveram 3,48 (IC95% 1,85–6,55) vezes a chance de serem nuligestas e 3,98 (IC95% 2,10–7,55) e 2,35 (IC95% 1,50–3,67) vezes a chance de nuliparidade e uma gestação, respectivamente; apresentaram 4,27 (IC95% 1,63–11,18) e 1,94 (IC95% 1,32–2,83) vezes a chance de ter nenhum filho e um ou dois filhos, respectivamente; apresentaram 1,57 (IC95% 1,11–2,22) vezes a chance de nunca terem fumado e 2,45 (IC95% 1,64–3,66) e 1,54 (IC95% 1,00–2,37) vezes a chance de serem diagnosticadas em estadiamento IA-IIA e IIB-IIIA, respectivamente.

**Tabela 3:** Razões de chances brutas das características epidemiológicas e clínicas na coorte de mulheres com diagnóstico de câncer cervical no Rio de Janeiro, segundo o tipo histológico.

Variável	AC	SCC	Teste X^2^	OR (IC95%)^c^
		
	n (%)	n (%)	p^b^	
Total	162 (16,1)	842 (83,9)		

Idade (anos)			1,00	
< 40	44 (16,1)	229 (83,9)		0,10 (0,68–1,46)
≥ 40	118 (16,1)	613 (83,9)		1
Cor da pele			0,927	
Branca	52 (15,9)	275 (84,1)		1
Não branca	110 (16,2)	567 (83,8)		1,03 (0,72–1,47)
Escolaridade			**0,002**	
≥ 12 anos de estudo	12 (30,8)	27 (69,2)		**3,01 (1,46–6,23)**
8 a 11 anos de estudo	83 (18,7)	361 (81,3)		**1,56 (1,10–2,21)**
< 8 anos de estudo	67 (12,9)	454 (87,1)		1
Estado conjugal			0,668	
Com companheiro	77 (15,6)	418 (84,4)		1
Sem companheiro	85 (16,7)	424 (83,3)		1,09 (0,78–1,52)
Menarca			0,918	
≥ 12 anos	116 (16,6)	584 (83,4)		1,05 (0,70–1,58)
< 12 anos	36 (15,9)	190 (84,1)		1
Idade da sexarca			**0,002**	
≥ 20 anos	40 (24,1)	126 (75,9)		**2,32 (1,43–3,77)**
17 a 19 anos	60 (18,9)	257 (81,1)		**1,70 (1,10–2,63)**
≤ 16 anos	40 (12,0)	292 (88,0)		1
Histórico de gestação			**< 0,001**	
Não	17 (38,6)	27 (61,4)		**3,48 (1,85–6,55)**
Sim	143 (15,3)	791 (84,7)		1
Número de gestações			**< 0,001**	
Nenhuma	17 (38,6)	27 (61,4)		**3,98 (2,10–7,55)**
1	33 (27)	89 (73)		**2,35 (1,50–3,67)**
> 1	110 (13,6)	696 (86,4)		1
Abortos			0,390	
Não	86 (16,6)	431 (83,4)		1
Sim	49 (14,4)	292 (85,6)		0,84 (0,57–1,23)
Número de abortos			**0,506**	
Nenhum	87 (16,6)	438 (83,4)		1,40 (0,80–2,43)
1	33 (15,5)	180 (84,5)		1,28 (0,68–2,41)
> 1	17 (12,5)	119 (87,5)		1
Número de filhos^a^				
Nenhum	7 (35,0)	13 (65,0)	**< 0,001**	**4,27 (1,63–11,18)**
1 a 2 filhos	78 (19,6)	319 (80,4)		**1,94 (1,32–2,83)**
> 2 filhos	52 (11,2)	412 (88,8)		1
Comorbidades			0,858	
Não	86 (16,3)	441 (83,7)		1
Sim	65 (15,8)	346 (84,2)		0,96 (0,68–1,37)
Tabagismo				
Nunca fumou	102 (18,9)	437 (81,1)	**0,012**	**1,57 (1,11–2,22)**
Fumante ou ex-fumante	59 (12,9)	397 (87,1)		1
Alcoolismo			0,142	
Nunca bebeu	88 (18,0)	402 (82,0)		1
Alcoolista ou ex-alcoolista	71 (14,5)	420 (85,5)		0,78 (0,55–1,09)
Estadiamento			**< 0,001**	
IA-IIA	63 (23,9)	201 (76,1)		**2,45 (1,64–3,66)**
IIB-IIIA	44 (16,5)	223 (83,5)		**1,54 (1,00–2,37)**
IIIB-IV	53 (11,3)	414 (88,7)		1

AC: adenocarcinoma cervical; SCC: carcinoma de células escamosas

^a^ Apenas em mulheres que gestaram.

^b^ p-valor dos testes do qui-quadrado ou exato de Fisher. Valores estatisticamente significativos (p < 0,05) em negrito.

^c^ Razão de chances (*odds ratio*) bruta.

Na análise de regressão logística múltipla, foi observado que o estadiamento IA-IIA esteve positivamente associado (OR = 1,79; IC95% 1,03–3,13) ao AC, independentemente da idade, escolaridade, idade da sexarca e número de filhos. De forma similar, a escolaridade maior ou igual a 12 anos de estudo (OR = 3,34; IC95% 1,27–8,76), não ter filhos (OR = 3,5; IC95% 1,27–9,85) ou ter até dois filhos (OR = 1,6; IC95% 1,02–2,50) também se mantiveram positivamente associadas ao AC, independentemente da idade e demais variáveis do modelo (Tabela 4).

**Tabela 4:** Razões de chances ajustadas e respectivos intervalos de confiança de 95% estimadas para a coorte de mulheres com câncer cervical, segundo o tipo histológico.

Variável	OR ajustada*	IC95%
Estadiamento		
IA-IIA	**1,79**	**(1,03–3,13)**
IIB-IIIA	1,00	(0,56–1,75)
IIIB-IV	1	
Escolaridade		
< 8 anos de estudo	1	
8 a 11 anos de estudo	1,27	(0,77–2,10)
≥ 12 anos de estudo	**6,30**	**(1,97–20,13)**
Idade da sexarca		
≤ 16 anos	1	
17 a 19 anos	1,38	(0,82–2,34)
≥ 20 anos	1,57	(0,82–3,03)
Número de filhos		
Nenhum	**3,81**	**(1,20–12,08)**
1 a 2	**1,74**	**(1,05–2,87)**
> 2	1	

-2Log-verossimilhança: 534,805*

* Razão de chances (*odds ratio*) ajustada para idade (contínua) e demais variáveis do modelo.

Valores estatisticamente significativos em negrito.

## DISCUSSÃO

Os resultados mostram que o tipo histológico mais frequente foi o SCC (83,9%), comparado ao AC (16,1%), corroborando outras pesquisas nacionais em que a frequência do AC variou entre 9,7% (estudo hospitalar realizado em Porto Alegre de 2005 a 2006^16^) e 12% (estudo realizado no Inca, no Rio de Janeiro, de 1999 a 2004^11^). Outros estudos de base hospitalar estimaram uma frequência que variou de 14% na Coréia do Sul (1988–2008)^17^ a 20% no Japão (2001–2010)^18^. Destaca-se que esses índices maiores procedem somente de países desenvolvidos e podem ser atribuídos a um melhor controle do câncer cervical do tipo escamoso nesses locais. Ao longo das últimas décadas, nos Estados Unidos e outros países desenvolvidos, tem havido uma redução constante da incidência do SCC e aumento da incidência do AC e suas variantes em termos relativos e absolutos, em função do aumento da detecção de lesões precursoras do SCC a partir da organização dos programas de rastreio. O mesmo não ocorre para o AC, muito provavelmente porque, conforme sugerido pelas pesquisas, a triagem baseada em citologia é mais eficaz para o rastreio das lesões precursoras do SCC.

Neste trabalho, foi observado que a média de idade ao diagnóstico não apresentou diferenças estatisticamente significativas entre os tipos histológicos AC e SCC no período estudado. Esses achados são consistentes com alguns estudos, que encontraram uma média de idade entre 47 anos (26–69 anos) na Itália entre 2003 e 2010^19^ e 53 anos (DP = 12 anos) no Japão entre 2001 e 2010^18^ para o AC e, para o SCC, uma média entre 47 anos (22–73 anos) em um estudo realizado no Japão entre 1984 e 2003^20^ e 51 anos (DP = 13) em outro estudo realizado no mesmo país entre 2001 e 2010^18^.

Ademais, o principal achado deste trabalho foi a associação positiva entre o AC e o estadiamento mais precoce (IA-IIA), sugerindo que as mulheres com AC apresentaram uma maior chance de serem diagnosticadas mais precocemente que as mulheres com SCC. Resultados similares foram encontrados num estudo retrospectivo de base populacional realizado nos EUA (1988–2005) com 24.562 mulheres diagnosticadas com estádios IB1 a IVB do câncer de colo uterino. Os autores observaram que as pacientes com AC apresentaram maior frequência da doença (26%) em estádio inicial (IB) no momento do diagnóstico em comparação com os 16,9% das mulheres com SCC para o mesmo estádio, reforçando que esses tipos histológicos são entidades clínicas distintas em sua forma de apresentação^21^. De modo semelhante, um estudo de coorte retrospectiva realizado nos EUA (1973–2002) verificou que a frequência das mulheres diagnosticadas com AC em estádio IB também foi maior que no tipo histológico SCC para o mesmo estádio (71% *versus* 51%, respectivamente)^3^. Do mesmo modo, um estudo hospitalar realizado no Brasil entre 2000 e 2009 utilizando informações de 239 hospitais mostrou que o estádio I foi mais frequente em mulheres diagnosticadas com AC (33,3%), enquanto 21,3% das mulheres apresentou SCC^12^. Isso demonstra que o perfil dessas mulheres é diferente e reflete as questões do acesso aos serviços de saúde e escolaridade.

Corroborando esses achados, nossos resultados também apontaram que as mulheres com AC exibiram maior nível de escolaridade que as mulheres com SCC. Além disso, a análise estratificada por tipo histológico e estadiamento revelou que as mulheres com AC e estadiamento I-IIA apresentaram maior nível de escolaridade (14,3%) comparadas àquelas com SCC (7,5%) (Tabela 2). Existem evidências que sugerem uma associação direta entre a escolaridade, o nível socioeconômico e o acesso aos serviços de saúde. O baixo nível de escolaridade, que geralmente é utilizado como uma variável substituta do nível socioeconômico, tem sido associado ao risco para o câncer cervical, sugerindo que essas mulheres podem não reconhecer a importância do exame ou não ter o conhecimento necessário para buscar rastreamento e tratamento, refletindo a falta de acesso aos serviços de saúde^22^. Esses achados são congruentes com o estudo de base hospitalar retrospectivo de Carmo e Luiz^11^ realizado no Inca (2003–2010), no qual foi observado que as mulheres com 11 anos ou mais de escolaridade apresentaram maior frequência de estádios I e II ao diagnóstico de câncer cervical, sendo que aquelas com o tipo histológico SCC foram mais comumente diagnosticadas em estádios mais avançados da Federação Internacional de Ginecologia e Obstetrícia – FIGO (p < 0,001), enquanto as mulheres com AC foram mais frequentemente diagnosticadas em estádio inicial. Essa associação pode ser explicada pelo fato de que mulheres com maior escolaridade geralmente buscam atendimento de saúde e têm mais conhecimento sobre prevenção, e assim sua doença é detectada em uma fase inicial, favorecendo um tratamento mais bem-sucedido^11^.

O estudo atual fornece evidência de uma associação positiva entre nuliparidade e AC. Esse achado é coerente com os resultados de um estudo de base hospitalar desenvolvido entre 1992 e 1996 numa população americana, com mulheres com SCC e AC^9^. Os autores observaram associação negativa entre quem gestou e o AC (OR = 0,4; IC95% 0,2–0,8) e associação positiva entre cinco ou mais gestações e o SCC (OR = 2,2; IC95% 0,9–5,4), sugerindo que os eventos reprodutivos, principalmente os relacionados à exposição endógena aos hormônios sexuais, podem ter efeitos diferentes sobre a progressão da infecção pelo HPV, afetando o tipo histológico do câncer cervical^9^. Pesquisas têm demonstrado que, além da infecção pelo HPV, que é a causa necessária para o câncer de colo uterino, os tipos histológicos AC e SCC compartilham outros fatores de risco, como o número de parceiros sexuais, idade da primeira atividade sexual, idade da primeira gestação e uso de contraceptivo oral^4,8,9,23^. O tabagismo e a multiparidade são fatores de risco diretamente associados ao SCC e inversamente associados ao AC^9,10^. Entretanto, alguns autores sustentam que o AC pode representar uma entidade histológica que compartilha dos fatores de risco relacionados ao câncer de endométrio, como a obesidade, o uso de anticoncepcional oral e a nuliparidade, além da associação inversa com o tabagismo^24^. Mulheres nulíparas têm maior número de ciclos menstruais ovulatórios devido à ausência de gravidez e lactação, com maior exposição cumulativa ao hormônio estrogênio e/ou menor exposição ao hormônio progesterona^24^. A progesterona afeta diretamente as células cancerígenas, causando inibição do crescimento das células neoplásicas e invasão celular^25^.

Em nosso estudo, foi observado ainda que, entre as mulheres com AC, a atividade sexual é iniciada mais frequentemente entre 17 e 19 anos de idade, enquanto as mulheres com SCC iniciaram a atividade sexual mais precocemente (≤ 16 anos). De modo semelhante, no estudo de base hospitalar que Altekruse et al.^9^ desenvolveram na população americana, foi observado que a idade precoce na primeira relação sexual (<17 anos *versus* ≥ 20 anos do grupo de referência esteve associada ao SCC (OR = 2,0; IC95% 1,0–3,9), mas não com o AC (OR = 0,9; IC95% 0,5–1,8). Isso sugere que outros fatores endógenos e ambientais, diferentes daqueles conhecidos para o SCC, poderiam atuar como cofatores modulando o risco de AC^9^. Em conjunto com as evidências da literatura, nossos achados sugerem que fatores ligados à exposição ao estrogênio endógeno, como a nuliparidade e ter até dois filhos, poderiam compor esse grupo de cofatores.

Nossos achados também indicaram que as mulheres com AC têm menor frequência de tabagismo, não havendo aumento do risco de AC entre as mulheres que fumavam. Achados semelhantes foram apresentados por Castellsagué et al.^4^, os quais fizeram uma análise conjunta de dados de estudos caso-controle de AC conduzidos e coordenados pela IARC em oito países (Argélia, Marrocos, Brasil, Peru, Paraguai, Índia, Tailândia e Filipinas) entre 1985 e 1997. Os autores não observaram associações estatisticamente significativas entre tabagismo e AC, embora tenham observado uma associação positiva entre tabagismo e SCC^4^. O tabagismo foi relatado por alguns autores^8,9^como fator de risco para SCC, mas não para AC. Esse achado é consistente com a hipótese de que as células glandulares são expostas a carcinógenos durante períodos de aumento da ectopia, a qual é menor em mulheres que fumam^26^. Além disso, foi relatado que a nicotina estimula o crescimento de células epiteliais em mulheres saudáveis^27^ e que também estimula o crescimento de células ectocervicais imortalizadas pelo HPV^28^. Desse modo, a falta de exposição aos carcinogênicos da fumaça do cigarro pela diminuição da ectopia e do aumento do crescimento das células ectocervicais pela nicotina poderiam ser responsáveis pela ausência de associação do tabagismo ao AC^23^.

Vale ressaltar que este estudo apresenta as limitações inerentes aos estudos retrospectivos com base em busca de prontuários, que nem sempre contêm dados completos. Dentre as limitações desta investigação, podem-se mencionar a ausência de exploração de algumas importantes exposições associadas ao AC apresentadas em outros estudos, como uso de anticoncepcional oral^9,23,29^, número de parceiros sexuais e idade da menopausa^30^. Além disso, trata-se de uma coorte hospitalar, não representando o que ocorre na coorte populacional do Rio de Janeiro. No entanto, esta pesquisa apresenta como vantagens o fato de ter sido a primeira no Brasil que avaliou a associação dos fatores de exposição entre os tipos histológicos AC e SCC, com a maior casuística do tipo histológico AC (n = 162) e com dados mais acurados por ter sido realizada uma busca não apenas no RHC, mas também diretamente nos prontuários.

## CONCLUSÃO

A frequência de AC observada neste estudo (16,1%) é semelhante às observadas em outros lugares do Brasil e do mundo. Quando comparadas às mulheres com SCC, aquelas com AC apresentaram maior escolaridade, sexarca mais tardia, nuliparidade ou máximo de dois filhos, menor frequência de tabagismo e predomínio do diagnóstico do câncer com estadiamento mais precoce. Os resultados encontrados destacam a diferença entre os tipos histológicos, sugerindo que o AC pode representar uma entidade clínica distinta de neoplasia cervical, podendo demandar abordagens distintas das para SCC. São necessários estudos com diferentes delineamentos epidemiológicos e maior tamanho amostral, visando testar as hipóteses relativas às associações entre os fatores de exposição aqui analisados e os diferentes tipos histológicos do câncer cervical.
